# The Close of the II. Volume

**Published:** 1869-04

**Authors:** 


### EDITORIAL DEPARTMENT.

*The Close of the II. Volume.*—With this Number we complete another Volume of the *American Journal of Dental Science*, the oldest publication of its class in the world.

Two years ago when we first entered upon the duties of an editor, in connection with our lamented colleague, we derived encouragement from the hope that many of the old patrons of this *Journal*, when it was so ably conducted by Professors Harris and Piggot, would gladly assist us in our efforts to revive a work which enjoyed so high a reputation in times past.

In this we have not been disappointed, and the words of encouragement and cheer we have received have been many, and coming from those whose opinions we regard as invaluable, we feel encouraged to persevere in our efforts to meet the expectations of our friends, and merit a continuation of the favor with which we have been rewarded for our labors during the past two years.

Indeed the success we have met with has far exceeded our expectations, and we feel gratified in being able to say at this time, that the *Journal* is now established upon so firm a basis as will ensure its future prosperity. Owing to this, and the fact that the *Journal*, through the enterprise of its Publishers, has now *a printing office* of its own, thereby materially lessening the cost of its publication, it has been determined to reduce the subscription price to $2.50 per annum, the postage to be paid by the subscriber. This reduction will place the work within the reach of all in the profession who may desire to obtain it, an object we have been striving for ever since we became connected with it.

